# Defective autophagy gets to the brain

**DOI:** 10.18632/oncotarget.6318

**Published:** 2015-11-13

**Authors:** Lorenzo Galluzzi, Guido Kroemer

**Affiliations:** Equipe 11 labellisée Ligue contre le Cancer, Centre de Recherche des Cordeliers, Paris, France; INSERM, U1138, Paris, France; Université Paris Descartes/Paris V, Sorbonne Paris Cité, Paris, France; Université Pierre et Marie Curie/Paris VI, Paris, France; Gustave Roussy Comprehensive Cancer Institute, Villejuif, France; Equipe 11 labellisée Ligue contre le Cancer, Centre de Recherche des Cordeliers, Paris, France; INSERM, U1138, Paris, France; Université Paris Descartes/Paris V, Sorbonne Paris Cité, Paris, France; Université Pierre et Marie Curie/Paris VI, Paris, France; Gustave Roussy Comprehensive Cancer Institute, Villejuif, France; Metabolomics and Cell Biology Platforms, Gustave Roussy Comprehensive Cancer Institute, Villejuif, France; Pôle de Biologie, Hôpital Européen Georges Pompidou, AP-HP, Paris, France; Department of Women's and Children's Health, Karolinska University Hospital, Stockholm, Sweden

**Keywords:** cerebral cavernous malformations, endothelial-to-mesenchymal transition, KRIT1, LC3, MTOR, p62

Macroautophagy (herein referred to as autophagy) is an evolutionary ancient mechanism that culminates in the lysosomal degradation of useless or potentially dangerous intracellular entities, be them endogenous (like damaged organelles) or exogenous (like invading bacteria). Autophagy not only contributes to the maintenance of cellular homeostasis in physiological conditions, but also plays an essential role in adaptation to stress. In line with this notion, defective autophagy has been etiologically implicated in a wide range of human pathologies, including cancer, inflammatory disorders and neurodegenerative conditions. Recent data demonstrate that autophagic defects also contribute to the development of genetic neurovascular conditions commonly known as cerebral cavernous malformations (CCMs).

CCMs (OMIM 116860, also known as cavernous angiomas or cavernomas) are neurovascular malformations that consist in clustered, aberrantly dilated and leaky capillaries lined up by a scarce endothelium and lacking normal structural components. CCMs can be inherited as a dominant autosomal disease with partial penetrance and variable expressivity or develop sporadically. Approximately 85-95% of familial CCM cases have been attributed to loss-of-function mutations in either of three genes: KRIT1, ankyrin repeat containing (*KRIT1*), cerebral cavernous malformation 2 (*CCM2*) and programmed cell death 10 (*PDCD10*). Mutations in unidentified genes or other hitherto unknown causes have been invoked to account for the remaining 5-15% cases of familial CCMs. In approximately 1/3 of cases, CCMs manifest clinically with moderate-to-severe symptoms including headaches, neurological deficits, seizures, strokes, and intracerebral hemorrhages. Of note, no treatment options other than the surgical resection of accessible lesions are currently available for subjects with clinically manifest CCMs [1].

Paolo Pinton and colleagues (from the University of Ferrara, Italy) have recently reported that various cell types subjected to the genetic inhibition of KRIT1 (by gene knockout or RNA interference) exhibit increased levels of two proteins that are normally processed (and degraded) by autophagy, *i.e*., sequestosome 1 (SQSTM1, best known as p62) and microtubule-associated protein 1 light chain 3 beta (MAP1LC3B, best known as LC3B) [2]. Such a defect was accompanied by the hyperactivation of mechanistic target of rapamycin (MTOR), a kinase with prominent autophagy-suppressing functions, and could be reversed (at least in part) by the reconstitution of KRIT1 activity as well as by the administration of two distinct pharmacological MTOR inhibitors [3, 4], namely, rapamycin and Torin 1. Similar data were obtained in *Pdcd10*^−/−^ cells. Moreover, the inability of KRIT1- deficient cells to properly process p62 and LC3 appeared to stem from defects in the late (rather than in the early) steps of autophagy, *i.e*., in the fusion of autophagosomes with lysosomes or in the lysosomal degradation of the autophagic cargo [5].

KRIT1-deficient cells exhibited molecular features of the so-called “endothelial-to-mesenchymal transition”, a phenotypic and biochemical shift that has previously been associated with CCMs [6]. Such molecular markers could be reversed by treating KRIT1-deficient cells with pharmacological MTOR inhibitors. Moreover, the small-interfering RNA (siRNA)-mediated depletion of an essential component of the autophagic machinery, i.e., autophagy-related 7 (ATG7), in KRIT1-proficient cells was associated with the appearance of both molecular and behavioral biomarkers of the mesenchymal state. Finally, in a mouse model of CCMs as well as in autoptic samples from CCM patients, endothelial cells from pathognomonic lesions (but not from the adjacent, normal brain) exhibited variable degrees of p62 accumulation [5].

Taken together, these data implicate defective autophagy in the pathogenesis of CCMs, and raise several important questions. First, what are the molecular mechanisms linking *KRIT1* loss-of-function mutations to autophagic defects? Second, do mutations in *CCM2* and *PDCD10* cause similar defects or do they operate at distinct levels of the autophagic cascade? Third, is the accumulation of p62 that drives CCM pathogenesis or does this reflect a generalized impairment in endothelial cell homeostasis? Fourth, and presumably most important, would the administration of MTOR inhibitors (some of which are currently approved by regulatory agencies as immunosuppressants) [7] benefit to CCM patients? Further experiments are required to solve these incognita.
